# Peer Reviewing for Early Career Faculty

**DOI:** 10.1063/4.0001097

**Published:** 2025-10-27

**Authors:** Kenneth C. Childers

**Affiliations:** California State University, Fullerton, Fullerton, CA, USA

## Abstract

Early career faculty members are a key resource in the peer review process, both in submitting manuscripts and reviewing others. Yet their exposure to the pipeline from submission to publishing relies heavily on their mentors in the early years. In this presentation, I will detail how early career faculty members can get a leg-up in peer review and, more importantly, how to balance this service with other important aspects in their career.

